# United States foreign aid freeze: An urgent call to action for support for African national laboratory programmes

**DOI:** 10.4102/ajlm.v14i1.2794

**Published:** 2025-05-16

**Authors:** Farouk A. Umaru

**Affiliations:** 1 Independent Laboratory Supply Chain and Quality Assurance Consultant, Washington, District of Columbia, United States

The United States government, under the Trump Administration, imposed a freeze on foreign aid by means of an Executive Order on 20 January 2025 (EO 14169. Reevaluation and Realigning United States Foreign Aid), initiating a 90-day pause on all foreign aid programmes. The United States Agency for International Development, established under the *Foreign Aid Act of 1961* (22 USC Ch. 32: Foreign Assistance),^1^ is an independent agency responsible for implementing United States foreign programmes and managing Congressionally allocated funds.

The President’s Emergency Plan for AIDS Relief is the largest aid initiative managed by the United States Agency for International Development, and is supported by the United States Centers for Disease Control and Prevention (CDC), Department of Defense, and State Department, significantly enhancing laboratory capacity in clinical diagnosis, disease surveillance and pandemic preparedness. The dismantling of the United States Agency for International Development’s operations will significantly affect laboratory services in many countries that rely on its health programmes. This opinion presents key historical contributions of the President’s Emergency Plan for AIDS Relief in strengthening the diagnostics landscape in Africa and makes the case that, if immediate action is not taken, the continent is on the verge of losing this progress, thereby reversing decades of success:

- **Procurement and supply chain of laboratory supplies and technologies:** For nearly 20 years, United States Agency for International Development implementing partners such as John Snow Inc. and Chemonics International have led the President’s Emergency Plan for AIDS Relief project in over 50 countries, delivering over $10 billion in health commodities, including HIV viral load tests, rapid diagnostic tests for HIV, tuberculosis, and malaria, antiretrovirals, and diagnostics equipment such as Cepheid GeneXpert, Abbott mPIMA, Roche Cobas instruments, Hologic Panthers, Abbott m2000, and Abbott mAlinity systems (these are distributed by United States companies significantly impacted by the Trump Administration’s America First Agenda).
- **Standardisation of diagnostic services:** Efforts by the United States CDC, the African Society for Laboratory Medicine, Africa CDC and partners have led to more than 400 laboratories receiving ISO 15189 and ISO 17025 accreditation,^2^ improving the quality of laboratory services on the continent. United States foreign aid programmes supported the procurement and technical assistance to these laboratories.
- **Disease surveillance and outbreak preparedness:** The Global Health Security programme, along with Africa CDC, the African Society for Laboratory Medicine, and the World Health Organization, has enhanced public health laboratory infrastructure in numerous countries, training thousands of staffers for disease detection and response, and assisting in the global surveillance and response to outbreaks that could arrive in the United States.
- **Harmonisation of tiered laboratory functions:** Over 10 African countries have restructured laboratories into a tiered network, aligning with the 2008 Maputo Declaration, optimising test menus, and staffing for efficiency and cost savings.^3^ The United States Agency for International Development has developed diagnostics networks optimisation strategies, supporting the restructuring of laboratory networks into an efficient and responsive turn-around time service delivery.^4^
- **Private sector participation in laboratory services:** The United States Agency for International Development has included private sector manufacturers, suppliers, and vendors in laboratory management, reducing risks of equipment downtime, stockouts, and expiration. The United States Agency for International Development negotiated all-inclusive agreements and service level contracts, and engaged local companies in last-mile vendor management for storage and distribution of essential laboratory supplies, improving access to site-level laboratory services.
- **Access to HIV viral load tests.** Achieving the Joint United Nations Programme on HIV and AIDS 95-95-95 goals (95% people living with HIV know their status, 95% of people who know their status initiate treatment, and 95% on treatment are virally suppressed) includes millions of people to be tested and virally suppressed.^5^ Over 50 million viral load test results have been delivered to 58 countries, accelerating countries’ progress towards the 95% targets of viral suppression, aiming to control the HIV pandemic by 2030.^6^
- **Controlling HIV/AIDS-related child mortality:** The early infant diagnostics service was expanded with modern multiplex technologies as a result of President Emergency Plan for AIDS Relief programme funding, allowing access to early detection and prevention of mother-to-child transmission.
- **Human resources for health:** Pre- and in-service training, curriculum reviews, and employment of expert personnel have strengthened national laboratory programmes and filled human capital gaps.
- **Local economic growth and stability:** Grants, sample distribution contracts, and technical assistance have created stable jobs for youth and supported local businesses.

Given the uncertainty surrounding the future of United States foreign aid, it is imperative for African leaders to take immediate action by mobilising resources to advance the continent’s laboratory programmes. Key considerations for immediate and sustainable laboratory programme resolutions are provided below:

- **Establish resilience funding:** African leaders and advisors should immediately mobilise resources to advance laboratory programmes across the continent. This includes setting up a laboratory resilience fund structure, modelled after the Global Fund or the Joint United Nations Programme on HIV and AIDS, to allow local and international philanthropies and other bodies to contribute towards laboratory programmes.
- **Increase domestic allocation:** African governments must increase financial commitments to diagnostics laboratory infrastructure, specifically clinical diagnosis, disease detection, surveillance, and response to clinical and emerging pandemics.
- **Engage private sector providers:** Africa CDC, the African Society for Laboratory Medicine, and related entities should engage the private sector in providing affordable essential testing services in countries where the private sector thrives. In addition, governments should incentivise private laboratory service providers through tax holidays for affordable services and support local manufacturing and supply chain infrastructure.
- **Partner with international companies:** Collaborate with international companies with bases in the United States, such as Abbott, Becton Dickinson, Hologic, Cepheid and Roche to continue with the current leasing and maintenance of service level agreements, and to encourage technology transfer for local production of diagnostics and pharmaceuticals, including patent waivers.
- **Foster local innovation:** Promote entrepreneurship within educational systems by providing incentives for science and technology startups, creating innovation hubs, and offering low-interest financing for emerging small businesses in laboratory technologies.
